# The unfolded protein response links ER stress to cancer-associated thrombosis

**DOI:** 10.1172/jci.insight.170148

**Published:** 2023-08-31

**Authors:** Oluwatoyosi Muse, Rushad Patell, Christian G. Peters, Moua Yang, Emale El-Darzi, Sol Schulman, Anna Falanga, Marina Marchetti, Laura Russo, Jeffrey I. Zwicker, Robert Flaumenhaft

**Affiliations:** 1 Division of Hemostasis and Thrombosis, Department of Medicine, Beth Israel Deaconess Medical Center, Harvard Medical School, Boston, Massachusetts, USA.; 2 Immunohematology and Transfusion Medicine, ASST Papa Giovanni XXIII, Bergamo, Italy.; 3 Hematology Service, Department of Medicine, Memorial Sloan Kettering Cancer Center, New York, New York, USA.

**Keywords:** Hematology, Cancer, Thrombosis

## Abstract

Thrombosis is a common complication of advanced cancer, yet the cellular mechanisms linking malignancy to thrombosis are poorly understood. The unfolded protein response (UPR) is an ER stress response associated with advanced cancers. A proteomic evaluation of plasma from patients with gastric and non–small cell lung cancer who were monitored prospectively for venous thromboembolism demonstrated increased levels of UPR-related markers in plasma of patients who developed clots compared with those who did not. Release of procoagulant activity into supernatants of gastric, lung, and pancreatic cancer cells was enhanced by UPR induction and blocked by antagonists of the UPR receptors inositol-requiring enzyme 1α (IRE1α) and protein kinase RNA-like endoplasmic reticulum kinase (PERK). Release of extracellular vesicles bearing tissue factor (EVTFs) from pancreatic cancer cells was inhibited by siRNA-mediated knockdown of IRE1α/XBP1 or PERK pathways. Induction of UPR did not increase tissue factor (TF) synthesis, but rather stimulated localization of TF to the cell surface. UPR-induced TF delivery to EVTFs was inhibited by ADP-ribosylation factor 1 knockdown or GBF1 antagonism, verifying the role of vesicular trafficking. Our findings show that UPR activation resulted in increased vesicular trafficking leading to release of prothrombotic EVTFs, thus providing a mechanistic link between ER stress and cancer-associated thrombosis.

## Graphical abstract

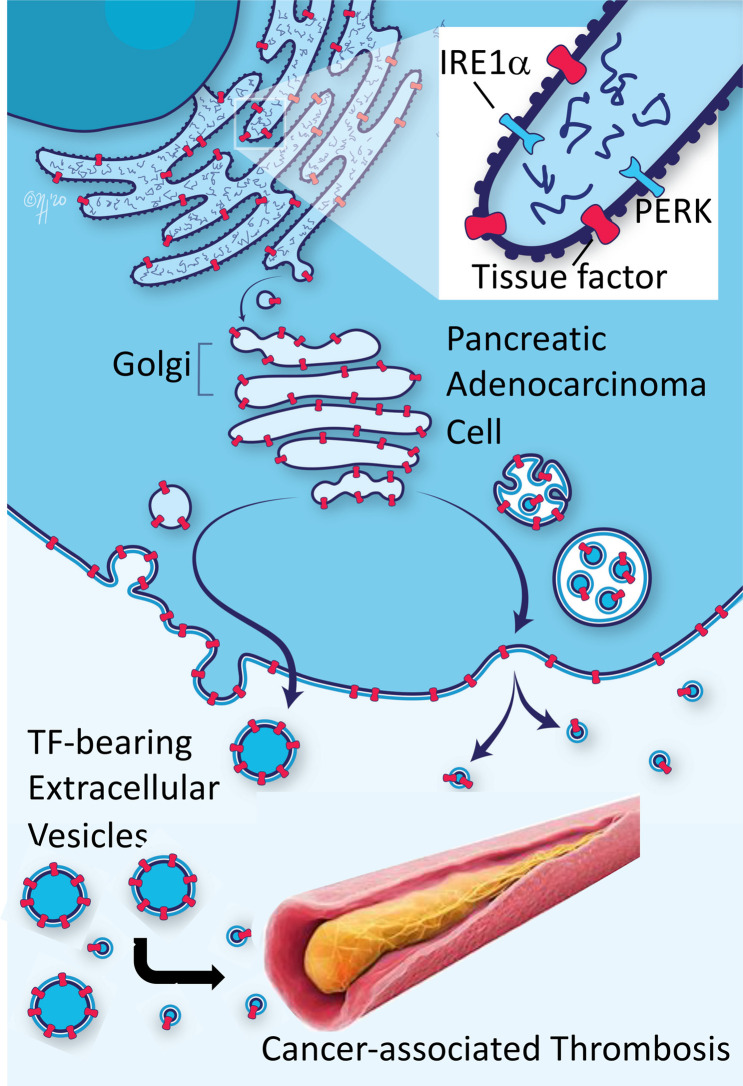

## Introduction

Thrombosis is a deadly and common complication of cancer. Both venous and arterial thrombosis are increased in the setting of malignancy (1–3). Nearly 10% of patients with cancer receiving outpatient chemotherapy die from thrombotic complications (4). The incidence of thrombosis in cancer ranges 4%–20% depending on several factors, including patient characteristics (e.g., age, sex, race, comorbidities), treatment-related factors (e.g., surgery, hospitalization, chemotherapy), and tumor-related factors (5, 6). One example of a tumor-related factor is the type of cancer, with brain, gastric, and pancreatic cancers posing the highest risk (6). Another important risk factor is stage of cancer, with advanced-stage cancer posing a greater risk (7). These observations underscore the importance of tumor-related characteristics in cancer-associated thrombosis (CAT) (8).

Progression of cancer is associated with activation of the unfolded protein response (UPR). Malignant transformation of pancreatic cancer to a more aggressive phenotype results from an anabolic switch with increased protein metabolism, enhanced accumulation of unfolded or misfolded proteins, and the activation of ER stress pathways, including the UPR (9, 10). The UPR increases the protein-folding capacity within the lumen of the ER in order to maintain proteostasis. This ER stress response includes upregulation of chaperone proteins to support protein folding, reduction of translational activity in order to reduce the quantity of unfolded proteins, and degradation of accumulated proteins through the ubiquitin/proteasome pathway (11, 12). The mechanisms are controlled by 3 ER receptors termed inositol-requiring enzyme 1α (IRE1α) (13, 14), protein kinase RNA-like endoplasmic reticulum kinase (PERK) (15, 16), and activating transcription factor 6α (ATF6α) (17). In quiescent, nonmalignant cells, these 3 receptors are bound by the abundant ER chaperone protein heat shock protein A5 (HSPA5; aka GRP78 or BiP). However, with the accumulation of unfolded or misfolded ER proteins in the setting of malignancy, HSPA5 is displaced from IRE1α, PERK, and ATF6α, and this displacement activates UPR pathways via these 3 receptors (18). The UPR also enhances lipid biogenesis, promoting expansion of endomembrane capacity and facilitating membrane trafficking (19–21). More recent studies have indicated that in addition to increasing the lipid component of cell membranes, the UPR enhances extracellular vesicle (EV) production (22–24).

While tumor-related mechanisms underlying the propensity for cancer to cause thrombosis are not well understood, the release of EVs expressing tissue factor (TF) has been invoked in both animal studies and clinical observations (25–27). TF expression is upregulated in many tumor cell lines and tumor biopsies (28–35). Yet the expression of TF in the tumor itself does not explain thrombosis at distant sites. The generation of TF-bearing extracellular vesicles (EVTFs) represents a mechanism whereby TF produced by a tumor can access the circulation. That cancer cells elaborate EVTFs has been recognized for more than 30 years (36). Several studies have shown that infusion of EVTFs derived from cancer cells into mice accelerates thrombus formation in vivo (37–39). Furthermore, clinical studies evaluating levels of EVTFs have demonstrated an association of elevated EVTF levels and venous thromboembolism (VTE) in the setting of cancer (40, 41).

Despite an association of tumor-derived EVTFs with CAT, the mechanisms of their production remain poorly understood. In particular, it is unknown whether ER stress results in increases in circulating prothrombotic EVTFs. Applying a prospective, proteomic approach using samples from the HyperCan study (42), we identified plasma proteins that were significantly increased in patients with cancer who subsequently developed VTE compared with those who remained free of VTE. Our analysis identified several proteins involved in the UPR. This finding prompted us to evaluate the role of the UPR in the elaboration of procoagulant EVs. These studies showed that activation of UPR receptors IRE1α and PERK triggered TF trafficking to the plasma membrane via an ADP-ribosylation factor 1–dependent (Arf1-dependent) mechanism. Enhanced TF trafficking along with increased EV generation resulted in the release of prothrombotic EVTFs following UPR induction. These studies identify potential prognostic markers to predict thrombosis in the setting of cancer and targets to prevent CAT.

## Results

### UPR-related plasma markers in CAT.

**Figure 1:**
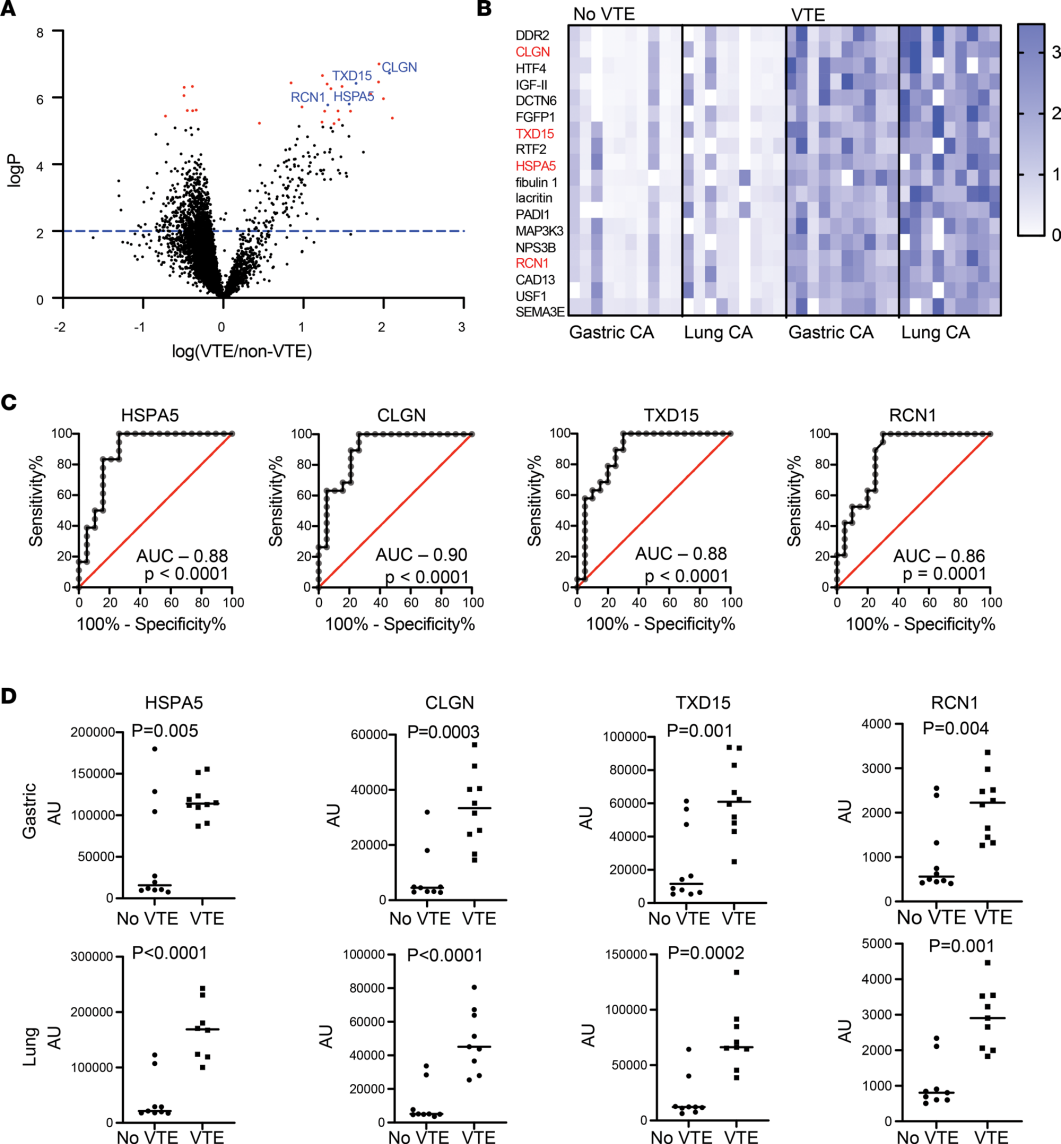
Plasma from patients with cancer who develop VTE is enriched in UPR-related analytes. Plasma samples were collected from 20 patients with gastric cancer and 19 patients with non–small cell lung cancer. Patients were followed prospectively for the development of VTE, which developed in 10 patients with gastric cancer and 9 patients with lung adenocarcinoma. (**A**) A volcano plot of 7,596 analytes tested using the SomaScan platform. Analytes that were significantly different (*P* < 6.6 × 10^–6^) between patients who developed VTE and those who did not are shown in red. Of these, 18 showed a greater than 2-fold difference between VTE and non-VTE, and the 4 shown in blue are UPR-related proteins. CLGN, calmegin; TXNDC15, thioredoxin domain containing 15 (or TMX5); RCN1, reticulocalbin 1. (**B**) The 18 proteins that were significantly elevated by >2-fold are shown in a heatmap that presents data for each patient normalized to the average value for that protein. Outliers are shown in white. (**C**) ROC curves of the 4 UPR-related proteins. (**D**) Values in patients who had no VTE over the observation period (no VTE) compared with those who went on to develop VTE (VTE) for UPR-related analytes for patients with gastric and non–small cell lung cancer are indicated (*P* values were obtained using a 2-tailed *t* test).

We evaluated plasma from 39 patients with advanced cancer enrolled in the HyperCan study (42) using Slow Off-rate Modified Aptamer (SOMA) technology (43, 44). Previous data from the HyperCan study revealed that biomarkers of hypercoagulability such as D-dimer and thrombin generation are predictive of disease progression and associated with shortened overall survival (45). The patients (20 with gastric cancer and 19 with non–small cell lung cancer) were followed prospectively for VTE. Among the patients analyzed, 10 with gastric cancer and 9 with lung cancer developed VTE (Supplemental Table 1; supplemental material available online with this article; https://doi.org/10.1172/jci.insight.170148DS1). To identify analytes among the 7,596 in the SomaScan panel that were significantly different between patients who developed VTE and those who did not, we selected proteins that showed ≥2-fold difference between non-VTE and VTE groups and an FDR-adjusted *P* < 0.05 (Figure 1A). This analysis identified 18 analytes that were upregulated in patients who subsequently developed VTE. No analytes that were downregulated in VTE met these criteria. Four of the 18 proteins (22%) were ER-resident proteins (Figure 1B). In contrast, ER proteins as defined in the Human Protein Atlas are not well represented in the SomaScan panel (<1% of total). In addition, all 4 ER-resident proteins identified have known functions in the UPR. HSPA5 (aka GRP78, 3-fold higher in the VTE cohort) is a critical regulator of the UPR that binds UPR receptors and maintains them in an inactivated state until displaced by unfolded or misfolded proteins (46–48). CLGN (increased 4.2-fold) is a functional analog of calnexin that is typically expressed in testes but is upregulated in malignancies, including gastric and non–small cell lung cancer. It forms complexes with protein disulfide isomerase family proteins, which function in thrombosis (49), and its expression is increased in the setting of ER stress (50–54). TXNDC15 (or TMX5, increased 3.2-fold) is a novel member of a family of membrane-bound thiol isomerases that facilitates disulfide bond formation in the ER and participates in ER stress responses (55, 56). RCN1 (increased 2.5-fold) is an ER protein that controls calcium homeostasis and is upregulated during the UPR as a protective mechanism (57–62). Evaluation of receiver operating characteristic (ROC) curves showed AUCs ranging from 0.86 to 0.90 for the 4 UPR-related analytes (Figure 1C). An advantage of our experimental design is the ability to analyze the predictive value of UPR-associated analytes separately in 2 distinct malignancies. Separate analysis of patients with gastric cancer and non–small cell lung cancer showed that all 4 of the analytes (CLGN, TXD15, HSPA5, and RCN1) were significantly increased in patients who developed clots in both gastric and non–small cell cancer cohorts (Figure 1D). These results suggest that activation of the UPR is associated with thrombosis in the setting of cancer, yet how UPR activation could promote CAT remains poorly understood.

### UPR induces TF activity in cancer cell supernatants.

**Figure 2:**
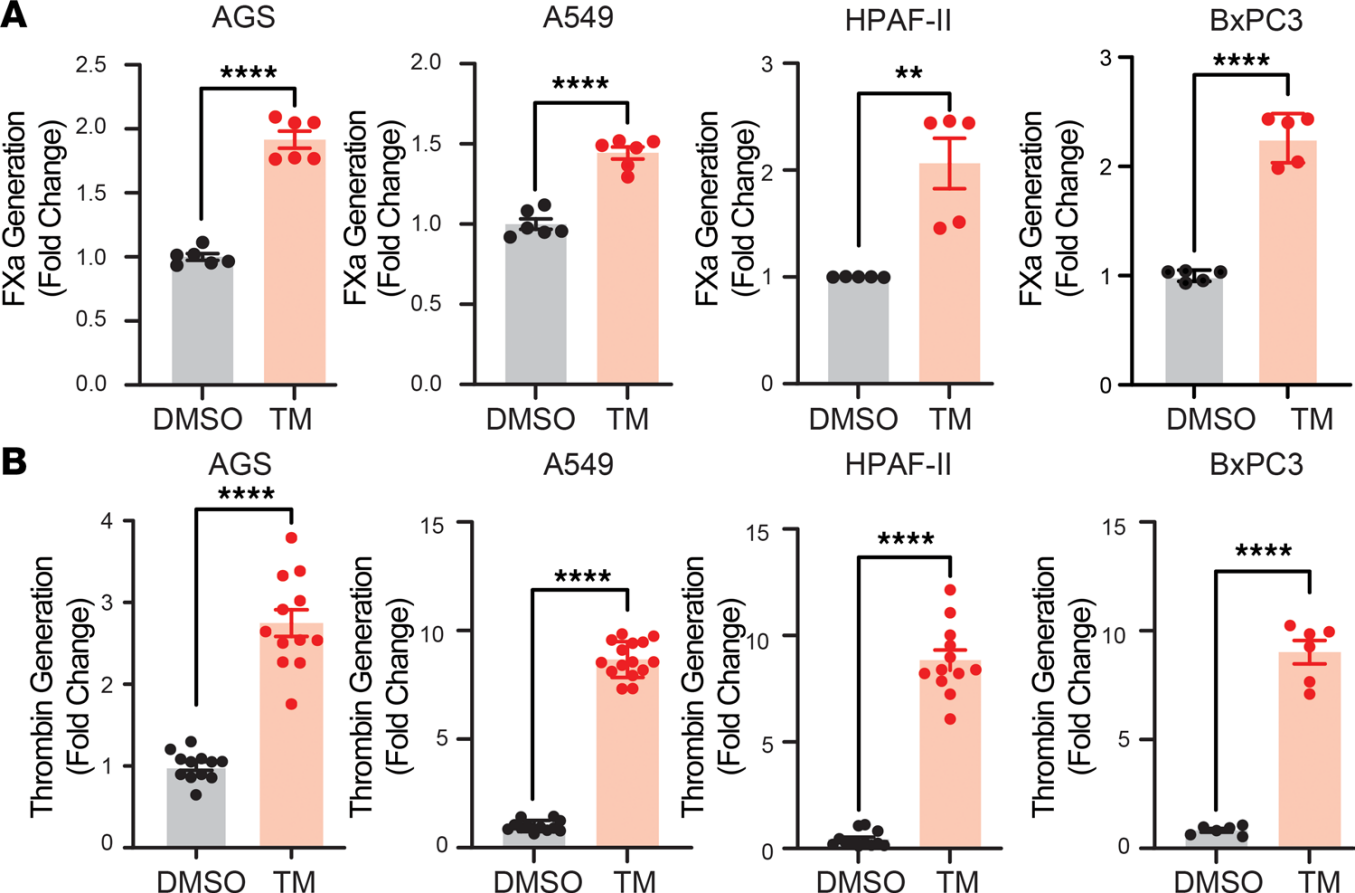
Induction of UPR results in increased TF activity in the supernatants of several adenocarcinoma cell lines. Human gastric cell adenocarcinoma (AGS), human lung adenocarcinoma cells (A549), and human pancreatic adenocarcinoma cells (HPAF-II and BxPC3) were exposed to 2.5 mg/mL tunicamycin for 4 hours. Supernatants were collected following this incubation, cleared, and subjected to serial centrifugation. The pellet was washed and evaluated for TF using a factor Xa generation assay (**A**) and thrombin generation (**B**). Thrombin generation was calculated based on the quantification of V*_max_* as described in Supplemental Methods. ****P < 0.0001, **P < 0.01 (*P* values obtained using a 2-tailed *t* test).

To assess the link between UPR induction and clinical thrombosis, we explored the possibility that induction of UPR in cancer cells results in the release of prothrombotic material. We evaluated AGS gastric cancer cells and A549 lung adenocarcinoma cells as well as HPAF-II and BxPC13 pancreatic cells, since both UPR activation (9, 63) and thrombosis (64, 65) are associated with pancreatic cancer. UPR was induced using tunicamycin, and supernatants were collected, cleared, and subjected to centrifugation as described in the Methods. The resultant pellets were analyzed for TF activity using a factor Xa (FXa) generation assay. UPR induction resulted in the generation of material that could be pelleted and possessed TF activity in the supernatants of all cell lines (Figure 2A). Procoagulant activity pelleted from supernatants also promoted thrombin generation (Figure 2B). Further studies focused on pancreatic adenocarcinoma cells since HPAF-II and BxPC3 showed high productivity of thrombin-producing activity in the supernatants and pancreatic adenocarcinoma is well known for its association with thrombosis.

### Characterization of EVs induced by the UPR.

**Figure 3:**
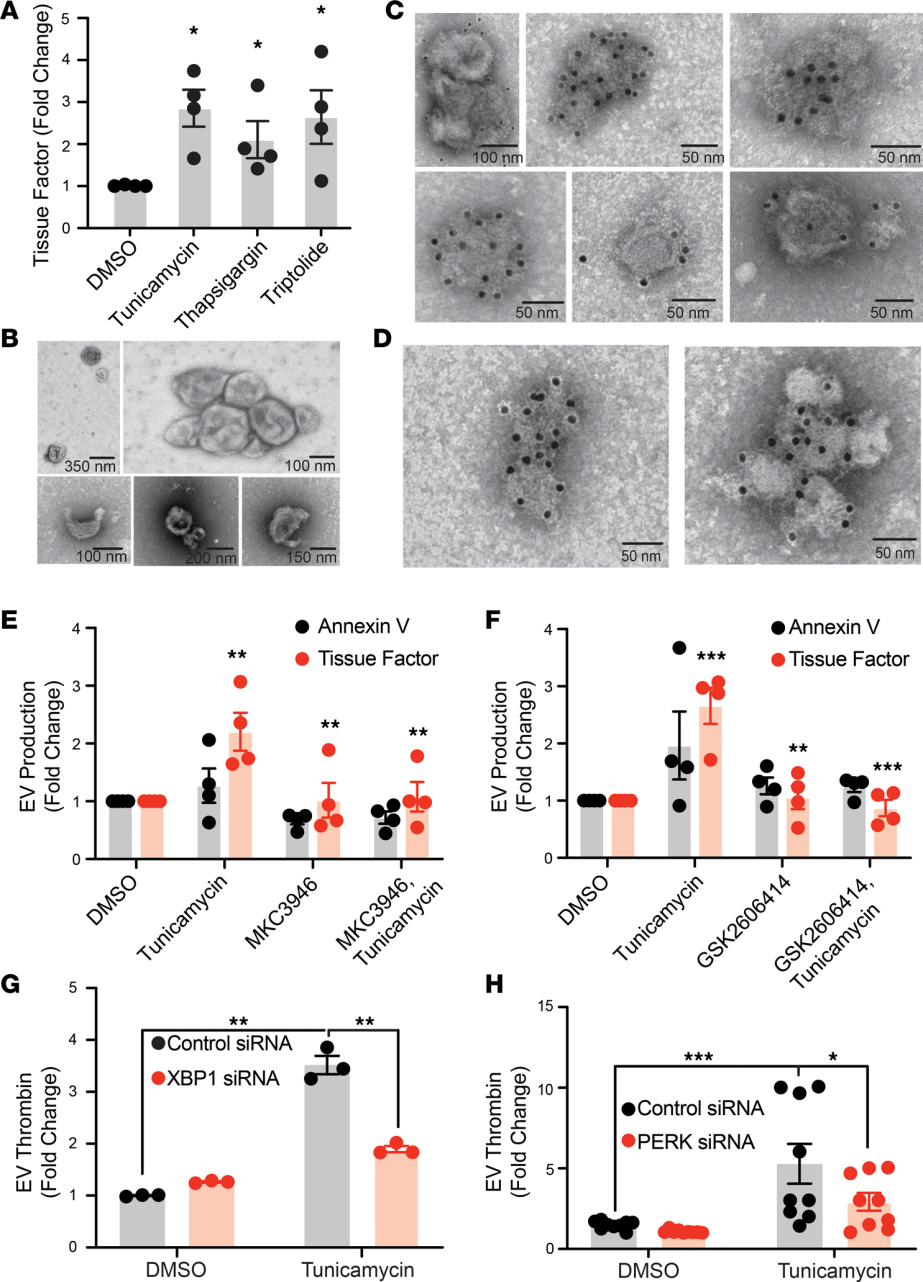
UPR induction in pancreatic cancer cells stimulates production of TF-bearing EVs. (**A**) HPAF-II cells were exposed to vehicle (DMSO), tunicamycin (2.5 mg/mL), thapsigargin (0.8 μM), or triptolide (0.2 μM) for 4 hours. Supernatants were collected and EVs isolated as described in the Methods. EVs were subsequently stained for TF and evaluated by flow cytometry. Error bars represent the mean ± SEM of 4 samples. *P < 0.01 (1-way ANOVA). (**B**) EVs isolated from HPAF-II cells following exposure to 2.5 mg/mL tunicamycin for 4 hours and evaluated using transmission electron microscopy (TEM). (**C** and **D**) EVs were generated and isolated as described in **B** and subsequently stained for CD9 (**C**) and TF (**D**). (**E** and **F**) HPAF-II cells were exposed to either 5 μM IRE1α inhibitor MKC3946 (**E**) or 1 μM of PERK inhibitor GSK2606414 (**F**) for 1 hour followed by 2.5 mg/mL tunicamycin for 4 hours. Supernatants were collected and EVs evaluated for binding of annexin V or anti-TF antibody using flow cytometry. Error bars represent the mean ± SEM of 4 samples. **P < 0.005, ***P < 0.001 (1-way ANOVA). Statistically significance differences were observed for EV TF expression between tunicamycin and DMSO, tunicamycin and MKC3946 or GSK2606414 alone, and tunicamycin alone and the presence of MKC3946 or GSK2606414 with tunicamycin. HPAF-II cells were exposed to 40 nM of either control siRNA or siRNA directed at (**G**) XBP1 or (**H**) PERK for 72 hours and subsequently exposed to vehicle or tunicamycin. EVs were isolated from supernatants and evaluated for thrombin generation. Error bars represent the mean ± SEM of 9 samples, *P = 0.01 (1-way ANOVA).

To assess the connection between UPR induction and TF release into supernatants, we used a variety of mechanistically independent chemical inducers of UPR, since tunicamycin, although widely used for induction of UPR, is not specific for UPR. Tunicamycin (causes accumulation of unfolded glycoproteins), thapsigargin (inhibits calcium-ATPase), and triptolide (causes downregulation of HSPA5) have all been shown to induce the UPR in HPAF-II cells (66–69). Exposure of HPAF-II cells to these UPR inducers resulted in 2.1- to 2.9-fold increases in TF-bearing material in the supernatants of HPAF-II cells (Figure 3A). Transmission electron microscopy (TEM) of the pelleted material isolated following induction of UPR by tunicamycin or triptolide revealed several distinct morphologies of EVs. Larger microvesicles were found in clusters or shapes reminiscent of helmet cells (Figure 3B). Smaller EVs (<100 nm) were also identified. These smaller EVs appeared in clusters and stained with antibody directed at CD9, suggesting that they were exosomes (Figure 3C). Staining with TF verified that EVs elaborated following induction of UPR-expressed TF (Figure 3D). Elaboration of TF-staining EVs was not limited to HPAF-II cells, since induction of UPR by tunicamycin also enhanced production of TF-bearing EVs by 2.4-fold ± 0.4-fold in BxPC3 cells, indicating that UPR-induced EVTF production is not cell line specific.

Both the IRE1α inhibitor MKC3946 (Figure 3E) and the PERK inhibitor GSK2606414 (Figure 3F) prevented tunicamycin-induced production of TF-bearing EVs. In contrast, neither MKC3946 nor GSK2606414 had a significant effect on the basal level of TF-bearing EV production. These findings suggest that UPR induction results in the release of TF-bearing microvesicles and exosomes from pancreatic adenocarcinoma cells. To verify the role of UPR receptors in the generation of procoagulant EVs from pancreatic adenocarcinoma cells, we evaluated the effect of X-box binding protein 1 (XBP1) and PERK depletion on EV-mediated thrombin generation using a plasma-based thrombin generation assay. IRE1α is a riboendonuclease that acts via the unconventional splicing of XBP1 mRNA (70, 71). Thus, XBP1 depletion inhibits the IRE1α/XBP1 pathway. Inhibition of this pathway using XBP1 siRNA had little effect on baseline levels of EV-mediated thrombin generation. However, thrombin generation from EVs obtained from HPAF-II after tunicamycin-induced UPR was reduced by 72% ± 10.7% (**P < 0.01) after XBP1 depletion (Figure 3G and Supplemental Figure 1A). PERK siRNA reduced EV-dependent thrombin generation by 51.1% ± 3.5% (*P* = 0.01) following tunicamycin-mediated induction of UPR (Figure 3H and Supplemental Figure 1B). The results were verified using a second siRNA directed at XBP1 (Supplemental Figure 2, A and C) and PERK (Supplemental Figure 2, B and D). Procoagulant activity in EVs generated by UPR induction in AGS gastric cancer cells and A549 lung cancer cells was inhibited by either MKC3946 or GSK2606414, further invoking the UPR in generation of procoagulant EVs (Supplemental Figure 3, A and B). Thus, inhibition of UPR reduces the elaboration of procoagulant EVs from adenocarcinoma cells.

### Procoagulant activity of EVs induced by the UPR.

**Figure 4:**
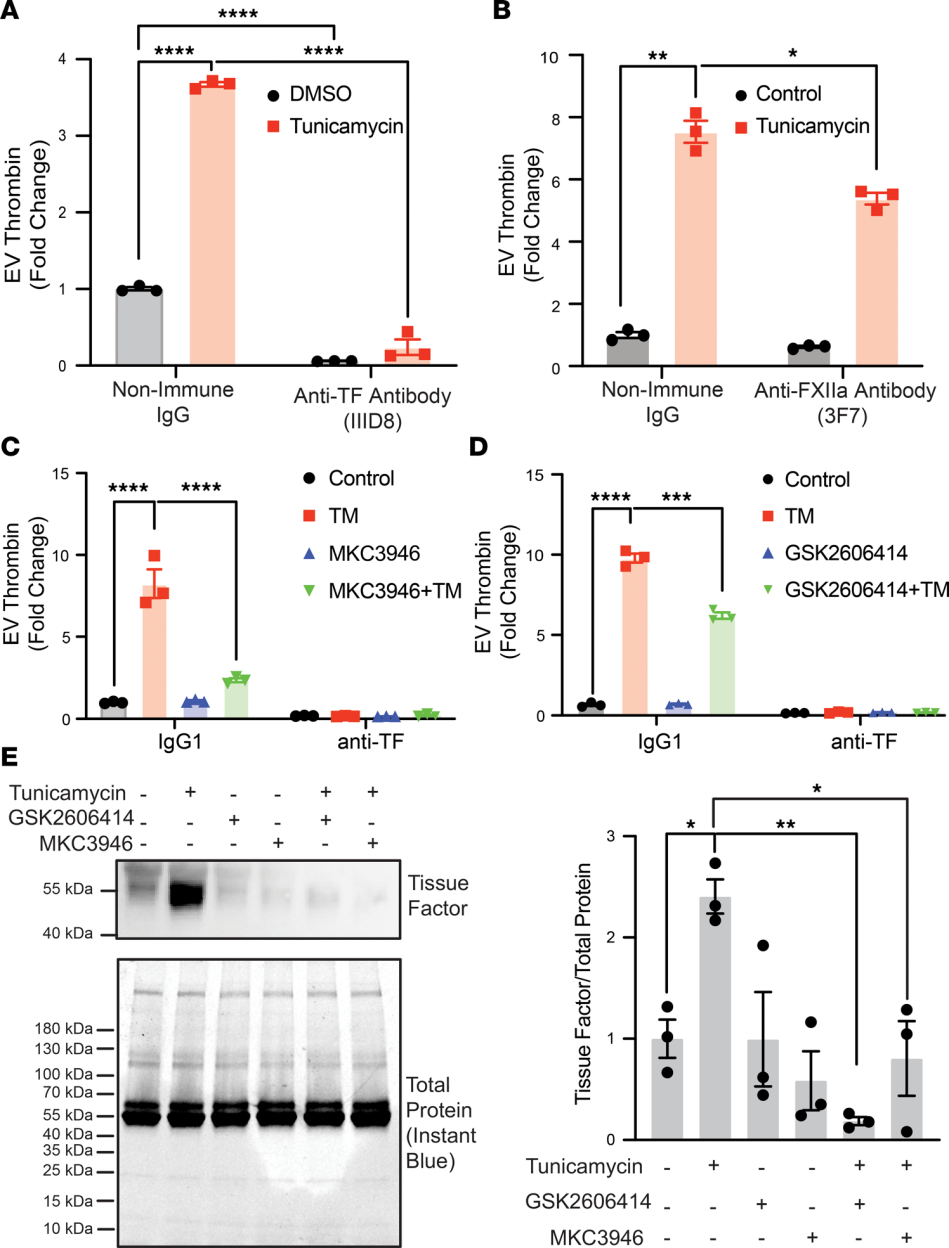
UPR induces procoagulant EVs from pancreatic cancer cells. (**A** and **B**) EVs were isolated from the supernatants of HPAF-II cells exposed to vehicle (DMSO) or 2.5 mg/mL tunicamycin. Isolated EVs were incubated with nonimmune IgG, (**A**) anti-TF antibody (IIID8), or (**B**) anti-FXIIa antibody (3F7) prior to evaluation of thrombin generation. Error bars represent the mean ± SEM of 3 samples, ****P < 0.0001, **P < 0.01, *P = 0.01 (1-way ANOVA). (**C** and **D**) BxPC3 cells were incubated with either 5 μM MKC3946 (**C**) or 1 μM GSK2606414 (**D**) for 4 hours and subsequently exposed to DMSO (Control) or 2.5 mg/mL tunicamycin (TM). EVs were isolated from supernatants and incubated with either nonimmune IgG or anti-TF antibody prior to evaluation of thrombin generation. Error bars represent the mean ± SEM of 3 samples, ****P ≤ 0.0001, ***P < 0.001 (1-way ANOVA). (**E**) HPAF-II cells were incubated with either 1 μM GSK2606414 or 5 μM MKC3946 for 1 hour prior to incubation with 2.5 mg/mL tunicamycin. EVs were isolated, lysed, and evaluated for protein concentration. Equal concentrations of proteins within EV lysates were subsequently separated by SDS-PAGE and analyzed for TF using Western blot analysis. Loading of total protein was assessed using Instant Blue (*left panel*). Quantification of 3 independent experiments (*right panel*). **P < 0.01, *P = 0.01 (1-way ANOVA).

Thrombin generation from tumor-derived EVs has been attributed to both the intrinsic (72) and the extrinsic pathways (73). To determine which was the dominant pathway of thrombin generation following induction of UPR, EVs were exposed to either anti-TF or anti-FXIIa antibodies prior to addition of plasma. While anti-TF antibody reduced thrombin generation by 95% ± 17% (*P* ≤ 0.0005; Figure 4A), anti-FXIIa antibody was less effective, reducing thrombin generation by 31% ± 3.1% (*P* = 0.01) (Figure 4B). Induction of UPR also increased the generation of procoagulant EVs from BxPC3 pancreatic adenocarcinoma cells (Figure 4, C and D). Procoagulant activity on BxPC3-derived EVs was decreased by inhibition of IRE1α by 71% ± 0.93% (*P* < 0.01; Figure 4C) or PERK by 37% ± 1.6% (*P* < 0.01; Figure 4D). Thrombin generation on BxPC3 EVs was also blocked completely by anti-TF antibody (Figure 4, C and D). Evaluation of EVs for TF by Western blot analysis verified that induction of UPR by tunicamycin increased the amount of TF delivered to EVs (Figure 4E). Inhibition of IRE1α or PERK using MKC3946 and GSK2606414 prior to UPR induction blocked the delivery of TF to EVs. These results indicate that the prothrombotic phenotype of EVs produced following UPR induction results primarily from TF activity.

### UPR-induced plasma membrane expression of TF in pancreatic adenocarcinoma cells.

**Figure 5:**
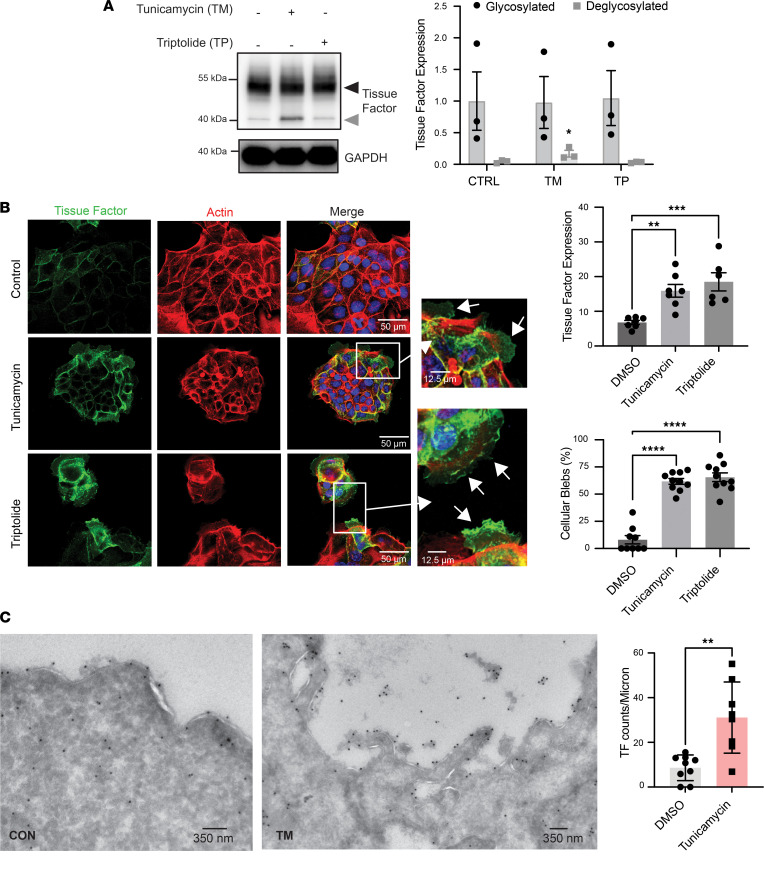
Induction of the UPR enhances cell surface TF expression. (**A**) HPAF-II cells were incubated in the presence of vehicle (DMSO), 2.5 mg/mL tunicamycin, or 0.2 μM triptolide for 4 hours. TF in cells was then analyzed by Western blot analysis (*left panel*) and quantified using densitometry (*right panel*). GAPDH was used as a loading control. Glycosylated (upper bands; black arrow) and deglycosylated (lower bands; gray arrow) TF were analyzed separately. *P = 0.01 (1-way ANOVA). (**B**) HPAF-II cells were exposed to DMSO (Control), 2.5 mg/mL tunicamycin, or 0.2 μM triptolide for 4 hours. Cells were then washed, fixed, permeabilized, and stained with antibody directed at TF (*green*), PE-phalloidin (*red*), and DAPI (*blue*). Cells were subsequently evaluated using 3-color immunofluorescence confocal microscopy. Arrows in magnified insets show TF-rich, actin-poor blebs. The graphs to the right represent the quantification of TF intensity and the percentage of cellular blebs as indicated. **P < 0.01, ***P < 0.001, ****P < 0.0001 (1-way ANOVA). (**C**) HPAF-II cells were grown on grids and subsequently exposed to vehicle (DMSO) or 2.5 tunicamycin for 4 hours. Cells were washed and fixed. Fixed cells were stained with anti-TF antibody (IIID8) followed by immunogold-labeled secondary IgG and evaluated by TEM as described in the Methods. The graph to the right shows quantification of TF on the cell membrane (gold particle number per micron of membrane). **P < 0.01 (2-tailed *t* test).

While induction of UPR in adenocarcinoma cells results in enrichment of TF in EVs, whether this enrichment results from increased TF production, trafficking of TF to plasma membrane, or both is not clear. To evaluate the production and redistribution of TF to EVs following induction of UPR, we first assessed cellular and EV-associated TF before and following UPR induction by tunicamycin or triptolide. Neither tunicamycin nor triptolide exposure significantly increased total TF levels as monitored by Western blot, though tunicamycin had the expected effect of inhibiting TF glycosylation as indicated by the increased intensity of the band representing nonglycosylated TF (Figure 5A, gray arrow). This observation raised the possibility that these UPR inducers caused increased trafficking of TF to the plasma membrane and EVs. We therefore performed confocal immunofluorescence (IF) microscopy to localize TF following induction of the UPR. Counterstaining with phalloidin to visualize actin and DAPI to visualize nuclei demonstrated clusters of HPAF-II cells (Figure 5B). TF localization to the plasma membrane was enhanced with UPR induction, increasing 2.3-fold ± 0.7-fold following exposure to tunicamycin and 2.7-fold ± 0.9-fold following exposure to triptolide. Of note, actin-poor, TF-rich blebs that emanated from the edges of the HPAF-II clusters were apparent following exposure to either tunicamycin or triptolide (Figure 5B, insets). To determine whether blebbing resulted from induction of apoptosis, we stained HPAF-II cells for cleaved caspase-3; however, very little staining was observed (Supplemental Figure 4). Evaluation of TF localization by TEM using immunogold staining showed increased TF at the cell surface following induction of the UPR (Figure 5C). An association of TF with membrane structures was visible on TEM (Figure 5C, quantification). These results indicate that following UPR induction, TF traffics from the plasma membrane to EVs.

**Figure 6:**
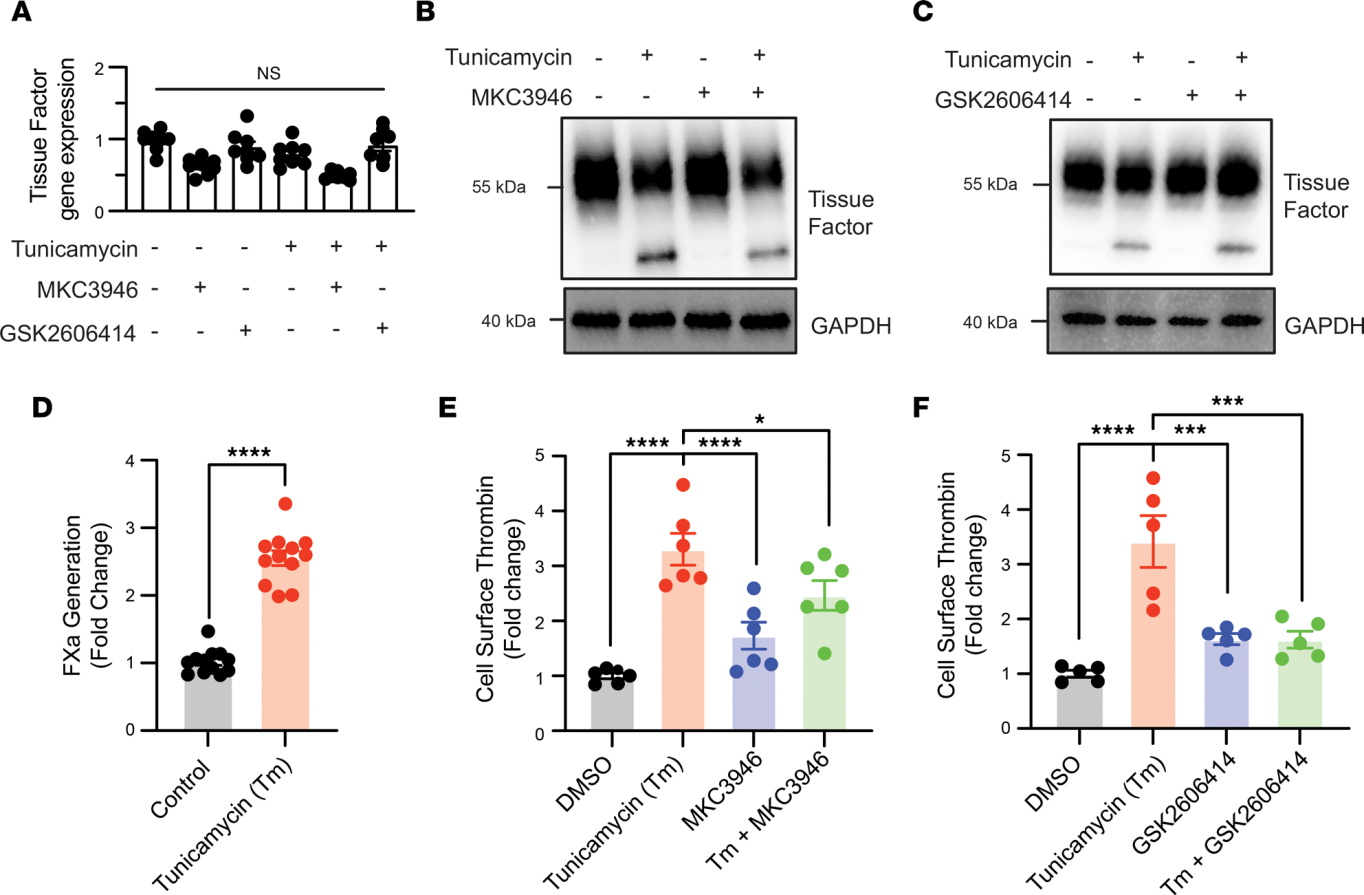
UPR does not mediate increased TF synthesis but promotes thrombin generation at the surface of pancreatic cancer cells. (**A**) HPAF-II cells were incubated with either 5 μM MKC3946 or 1 μM GSK2606414 for 1 hour prior to exposure to 2.5 mg/mL tunicamycin or vehicle (DMSO) for 4 hours. After a 1-hour incubation, cells were lysed and TF transcript levels quantified using quantitative PCR. (**B** and **C**) HPAF-II cells were incubated in the presence of vehicle. *Not significant* (1-way ANOVA). (**B**) MKC3946 or (**C**) GSK2606414 for 1 hour prior to stimulation with either vehicle (DMSO) or 2.5 mg/mL tunicamycin for 4 hours. TF in cells was then analyzed by Western blot analysis. (**D**) HPAF-II cells were exposed to 2.5 mg/mL tunicamycin for 4 hours. The supernatant was removed, cells were washed, and cells’ surface factor Xa (FXa) activity was evaluated using a FXa assay as described in Supplemental Methods. Error bars represent the mean ± SEM of 3 samples, **P < 0.005, ***P < 0.001, ****P < 0.0001 (1-way ANOVA). (**E** and **F**) HPAF-II cells were exposed to either 5 μM MKC3946 (**E**) or 1 μM GSK2606414 for 1 hour (**F**) followed by 2.5 mg/mL tunicamycin for 4 hours. The supernatant was removed, cells were washed, and thrombin generation on cells’ surfaces was evaluated. Error bars represent the mean ± SEM. ****P ≤ 0.0001, ***P < 0.0005, *P = 0.01 (1-way ANOVA).

To more rigorously evaluate the question of whether UPR induces upregulation of TF production or trafficking of TF to plasma membrane, we assessed the effect of UPR inhibitors on *F3* transcript, TF protein levels, and FXa generation in HPAF-II cells. The UPR inhibitors MKC3946 and GSK2606414 did not significantly affect *F3* gene expression (Figure 6A). Similarly, inhibitors of UPR did not affect expression levels of TF (Figure 6, B and C, and Supplemental Figure 5, A and B). Despite the lack of effect of UPR on total TF levels, UPR induction increased TF activity on the surface of HPAF-II cells by 2.7-fold ± 0.06-fold (*P* < 0.0001) as measured using a FXa generation assay (Figure 6D), which is an indicator of TF function on the cell surface. Induction of the UPR also increased TF activity (Figure 6E). Inhibition of IRE1α resulted in substantial inhibition of UPR-induced thrombin generation on the pancreatic cell surface (53.5% ± 11.1%, *P* < 0.02; Figure 6E), while blocking PERK resulted in complete inhibition (99.6% ± 5.3%, *P* < 0.001; Figure 6F). Overall, these studies show that UPR induction does not cause an increase in TF production, but rather stimulates increased cell surface expression and activity of TF, rendering the cell surface procoagulant.

**Figure 7:**
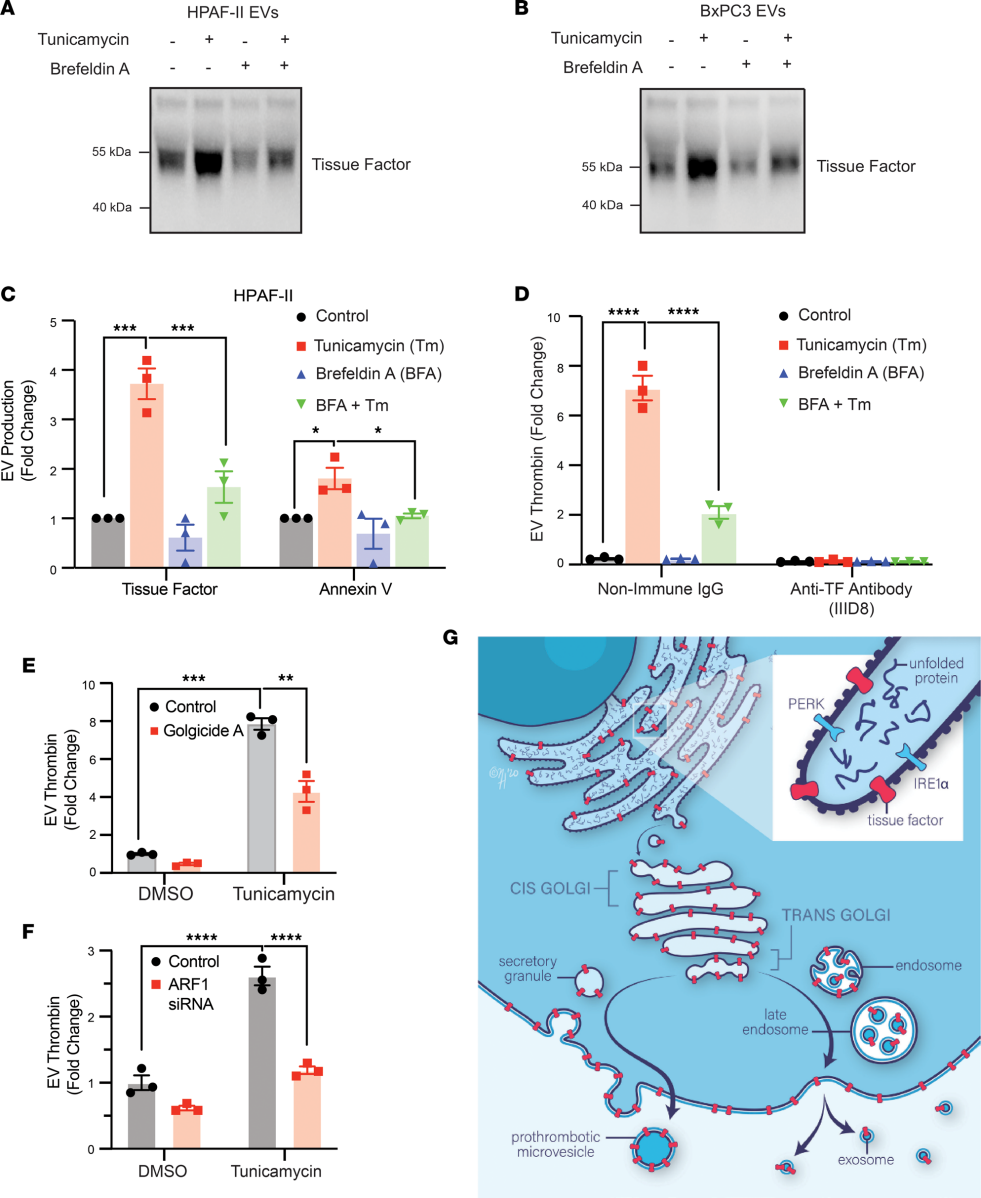
UPR-induced production of procoagulant EVs involves ER to Golgi transport. (**A**) HPAF-II or (**B**) BxPC3 cells were incubated with vehicle (DMSO) or brefeldin A for 1 hour prior to stimulation with vehicle (DMSO) or 2.5 mg/mL tunicamycin for 4 hours before analysis of TF in EVs. (**C**) HPAF-II cells were exposed to 3 μM brefeldin A for 1 hour followed by 2.5 mg/mL tunicamycin or DMSO for 4 hours. EVs were isolated from supernatants and evaluated using annexin V or anti-TF antibodies by flow cytometry. Error bars represent the mean ± SEM of 3 samples, *P < 0.01, ***P < 0.001 (1-way ANOVA). (**D**) HPAF-II cells were exposed to 3 μM brefeldin A for 1 hour followed by 2.5 mg/mL tunicamycin or DMSO for 4 hours. EVs were isolated from supernatants and incubated in the presence of nonimmune IgG or IgG directed at TF (IIID8). Samples were subsequently evaluated for thrombin generation. Error bars represent the mean ± SEM of 3 samples, ****P ≤ 0.0001 (1-way ANOVA). (**E**) HPAF-II cells were exposed to 1.5 μM Golgicide A for 1 hour then 2.5 mg/mL tunicamycin or vehicle (DMSO) for 4 hours. EVs were isolated from supernatants and subsequently evaluated for thrombin generation. Error bars represent the mean ± SEM of 3 samples, **P < 0.01, ***P < 0.001 (1-way ANOVA). (**F**) HPAF-II cells were exposed to 40 nM of either control siRNA or siRNA directed at Arf1 for 48 hours and subsequently exposed to either DMSO or 2.5 mg/mL tunicamycin for 4 hours. EVs were isolated from supernatants and evaluated for thrombin generation. Error bars represent the mean ± SEM of 3 samples, ****P ≤ 0.0001 (1-way ANOVA). (**G**) Schematic model of TF trafficking to the cell surface and to EVs following activation of the UPR in pancreatic adenocarcinoma cells. Increased protein translation with malignant transformation results in increased abundance of unfolded proteins, activation of ER stress receptors, and increased TF trafficking.

TF trafficking to the cell surface and subsequent incorporation into membrane blebs appears to be a major mechanism of EVTF generation following UPR induction. This observation raises the question of how TF traffics to extracellular locales. EVs including exosomes can be generated through nonclassical pathways that do not require ER to Golgi transport (74–77). In addition, TF could redistribute exclusively from post-Golgi compartments. On the other hand, TF typically traffics through the Golgi apparatus, where it is glycosylated, even though glycosylation is not essential for its transit through the Golgi (78). To assess whether TF traffics to EVs through the classical pathway or from post-Golgi compartments, we used brefeldin A, a small molecule that blocks ER to Golgi transport and promotes Golgi disassembly by inhibiting a guanine nucleotide exchange factor, GBF1 (79, 80). Incubation with brefeldin A prior to induction of UPR by tunicamycin reduced TF in EVs derived from both HPAF-II (Figure 7A and Supplemental Figure 6A) and BxPC3 cells (Figure 7B and Supplemental Figure 6B). Evaluation of EVTFs by flow cytometry showed that incubation with brefeldin A prior to induction of UPR significantly reduced the generation of EVTFs (Figure 7C). Brefeldin A also inhibited the TF-dependent procoagulant activity of EVTFs generated by induction of the UPR (Figure 7D). Inhibition of procoagulant activity occurred despite the fact that brefeldin A stimulated *F3* gene expression 1.5-fold by itself and 1.7-fold in the presence of tunicamycin (Supplemental Figure 6C). Brefeldin A did not significantly affect TF protein levels (Supplemental Figure 6D). A second, structurally distinct inhibitor of GBF1, termed Golgicide A, also blocked UPR-mediated generation of procoagulant EVTFs (Figure 7E). GBF1 is an exchange factor for the GTPase Arf1, which is required for COPI complex formation at the Golgi (81). Since GBF1 activates Arf1, we evaluated the effect of Arf1 knockdown on procoagulant EV production following UPR induction. Arf1 levels following exposure of HPAF-II cells to siRNA directed at Arf1 were 43% ± 3.5% of controls (Supplemental Figure 1C). Knockdown of Arf1 by siRNA inhibited EV-dependent thrombin generation by 88% ± 5.7% (*P* < 0.0001) (Figure 7F). Taken together, our studies support a model whereby induction of UPR results in loss of ER proteostasis, resulting in enhanced vesicular transport through the Golgi and generation of TF-bearing exosomes and microvesicles (Figure 7G).

## Discussion

The finding that UPR-related plasma proteins were elevated in patients with cancer who developed VTE compared with those who did not prompted us to evaluate the connection between UPR activation and thrombosis in the setting of adenocarcinoma. ER proteins as defined by the Protein Atlas are not well represented in the SomaLogic analyte panel, representing <1% of analytes. In contrast, >20% of proteins found to be significantly elevated by >2-fold in VTE compared with non-VTE patients were ER proteins with roles in the UPR (Figure 1). Although the increase in levels of these markers needs to be confirmed in larger cohorts using validated ELISAs, ROC curve analyses raise the possibility that evaluation of UPR markers in cancer could provide prognostic information to help guide thrombosis risk assessment and the use of anticoagulation. These markers were significantly elevated (Figure 1) in both gastric and non–small cell lung cancer, suggesting that such proteins could be used broadly as prognostic markers in adenocarcinoma. This putative association of UPR-related proteins with VTE in the setting of cancer invites the question of whether these UPR markers are derived from the malignancy or unaffected tissue. HSPA5 and RCN1 are substantially elevated in cancer, and their levels associate with progression of malignancy (82, 83). CLGN is considered a testis-specific protein in the healthy host but is upregulated in cancer (54). These considerations point to the tumor as a source of elevation of UPR markers. However, further studies will be required to determine the origin of these UPR markers.

Thrombosis in the setting of cancer often occurs at sites distant from the site of tumor, implicating circulating factors as an etiology for CAT. Among the most well-characterized circulating procoagulant factors in cancer are EVTFs (40, 84). We have targeted 2 major UPR receptors to assess the role of the UPR in the generation of procoagulant EVs. Inhibition of either IRE1α/XBP1 or PERK pathways blocks UPR-mediated generation of procoagulant EVs from adenocarcinoma cells (Figures 2–4 and Supplemental Figure 2). Knockdown of XBP1 or PERK also decreases UPR-mediated generation of procoagulant EVs (Figure 3). Previous studies have shown that TF is expressed on the surface of many tumors, including pancreatic, non–small cell lung, and gastric cancer (31–35). This surface TF can be transferred to EVTFs through the formation of microvesicles (84, 85). Consistent with the premise that TF on the surface of these EVs mediates their procoagulant activity, anti-TF antibody completely blocks thrombin generation mediated by UPR-induced EVs, whereas anti-FXIIa antibody has only a modest effect. The UPR can promote either cell survival or apoptosis depending on the context and extent of UPR activation, complicating the interpretation of results since procoagulant EVs can be released during apoptosis. However, staining HPAF-II cells for cleaved caspase-3, a marker of apoptosis, did not demonstrate apoptosis even following induction of the UPR under the same conditions that caused EVTF generation (Supplemental Figure 3). An alternative possibility is that UPR survival pathways in cancer promote enhanced vesicular trafficking of TF and stimulate release of EVTFs.

Our results indicate that the production of EVTFs following induction of UPR in adenocarcinoma cells involves TF trafficking through classical ER/Golgi pathways. Previous studies in nonmalignant cells including fibroblasts and endothelial cells show that TF traffics through Golgi, and indeed a significant reservoir of TF resides in the Golgi (86–88). The disassembly of the Golgi by brefeldin A or Golgicide A in pancreatic adenocarcinoma cells may both prevent its trafficking and cause the displacement of an important pool of TF. Our studies suggest a working model (Figure 7G) whereby induction of the UPR results in increased trafficking of TF through classical ER/Golgi pathways and distribution to both the plasma membrane and EVs. Inhibition of UPR-induced procoagulant EV production by *ARF1* siRNA supports this assertion (Figure 7). Following transit through Golgi, TF is delivered to the plasma membrane and EVs. IF microscopy and TEM show blebbing of TF-rich, actin-poor plasma membrane (Figure 5), which could give rise to TF-bearing microvesicles. Consistent with this interpretation, evaluation of microvesicles and exosomes released following induction of UPR demonstrate that they are enriched for TF (Figures 3 and 4). Several stimuli including inflammatory cytokines (e.g., TNF-α) (89), hypoxia (90), growth factors (91), and oncogenic mutations (92) enhance TF transcription and translation, ultimately leading to increased TF surface expression. Further studies will be required to determine whether ER stress contributes to increased surface trafficking of TF and EVTF formation in response to these stimuli and, if so, which of the UPR pathways — IRE1α, PERK, or ATF6 — is involved in these processes.

A second component of the generation of procoagulant EVs is the increase in overall EV formation stimulated by UPR induction. The UPR serves an essential role in linking upregulation of protein-folding capacity to lipid biogenesis (19–21, 93, 94). Activation of UPR can result in massive ER expansion (95, 96) and is an important mediator of lipid metabolism in cancer (97). Stimulation of the IRE1α/XBP1 pathway, for example, initiates phosphatidylcholine synthesis through upregulation of choline kinases, phosphocholine cytidylyltransferases, and cholesterol production (98–100). UPR induction can also lead to upregulation of phosphatidylethanolamine (101). With regard to phosphatidylserine (PS), oxysterol-binding homology proteins, ORP5 and ORP8, transport PS from ER to the plasma membrane in the setting of ER stress and control PS levels at the plasma membrane in pancreatic cancer (102–104). PS expression is required for efficient activation of the coagulation cascade and generation of EVs (105–107). Thus, PS expression could enhance the TF activity observed both on cancer cell surfaces and on EVTFs. Although a limitation of our study is that we did not evaluate UPR-induced PS exposure comprehensively, our initial evaluation demonstrated a trend toward inhibition of PS exposure with blockade of UPR (Figure 3). UPR-mediated elaboration of EVs has been observed in several cell types, including hepatocytes (108), smooth muscle cells (23), and pancreatic β cells (109). Our studies show that inhibition by brefeldin A inhibits UPR-mediated EV generation (Figure 7). That inhibition of ER to Golgi transport blocks EVTF production was not entirely anticipated since secretion of EVs does not necessarily proceed through classical ER/Golgi pathways and can be resistant to brefeldin A (74–77). However, in other systems, inhibitors of brefeldin A–sensitive ARF-guanine exchange factor block EV release (110, 111). Whether de novo phospholipid biogenesis initiated by UPR induction in the setting of cancer results in altered phospholipid composition in plasma membrane and perhaps EVs is an area of future investigation. This possibility is intriguing in the context of CAT since TF activity is markedly influenced by membrane phospholipid content (112, 113).

Although our study identifies a link between the UPR and clot formation in CAT, it has several limitations. HSPA5, RCN1, CLGN, and TXD15 need to be validated using an assay method compatible with use in a central lab (e.g., ELISA) in a large prospective study of patients with advanced-stage cancer before these proteins can be used to guide anticoagulation prophylaxis. Some of these markers are ubiquitous among cells and cancer types (e.g., HSPA5), but others may be useful only for specific malignancies (e.g., CLGN). With regard to mechanism of the UPR in promoting cancer, simultaneous evaluation of EVTF and/or TF procoagulant activity along with validated UPR markers will be useful in assessing the hypothesis that UPR contributes to CAT by stimulating the generation of EVTF. In addition, although our work has focused on EVTFs because of their well-studied association with CAT, the UPR involves extensive reprogramming of cells and is likely to contribute to thrombosis in cancer via a variety of mechanisms in addition to EVTF generation.

In summary, we show that activation of the UPR in cancer promotes generation of prothrombotic EVTFs. Specifically, our studies demonstrate roles for both IREα/XBP1 and PERK pathways in the formation of EVTFs (Figure 7G). This pathway provides a potential connection between ER stress and VTE. Future studies using animal models will be required to confirm a causal relationship of these pathways in thrombus formation in the setting of cancer. Nonetheless, both murine models and clinical studies have linked plasma EVTF levels to CAT, particularly in the setting of pancreatic cancer (37, 40, 84, 114–116). Our proteomics evaluation of VTE in patients with cancer suggests clinical implications of our findings. Prospective validation of the association between UPR and VTE could lead to improved diagnostics for assessing thrombotic risk in cancer. Furthermore, components of the UPR might represent novel therapeutic targets in CAT. Inhibition of PERK using GSK2606414 blocks thrombus formation in vivo in a FeCl_3_-induced thrombosis model as well as in a restenosis model (117, 118). Further elucidation of mechanisms through which activation of UPR in cancer cells increases the likelihood of thrombosis may ultimately lead to novel prognostic and therapeutic strategies to mitigate risk to patients of this serious (and sometimes fatal) complication of malignancy.

## Methods

### Patient samples and proteomic analysis.

Plasma samples were analyzed from 39 patients with gastric or non–small cell lung cancer as part of the HyperCan study (42). Eligibility for enrollment included Eastern Cooperative Oncology Group performance status of 0–2, life expectancy > 3 months, and eligible for chemotherapy treatment. Patients were excluded if they were receiving anticoagulant therapy (42). Blood samples were obtained at baseline prior to cancer therapy. Fasting peripheral venous blood samples were collected into 6 mL Vacutainer tubes containing 0.109 M Na3 citrate (9:1 v/v; Becton Dickinson). Platelet-poor plasma was obtained by double centrifugation at 2,600*g* for 15 minutes at 25°C and stored at −80°C. Patient characteristics are included in Supplemental Table 1. Candidate UPR biomarkers were analyzed by SomaScan (SomaLogic), which is a commercial high-throughput proteomics platform of modified aptamers to quantify plasma proteins (43, 44). A library of 7,596 fluorescence-labeled SomaScan aptamers, coupled with a photocleavable linker and biotin, were used for evaluation of plasma proteins as previously described (119).

### Isolation of EVs from cell culture.

EVs were isolated from the supernatant of pancreatic cancer cell lines HPAF-II and BxPC3, lung cancer cell line A549, and gastric cancer cell line AGS (ATCC) after treatment with UPR inducers, UPR inhibitors, and/or inhibitors of vesicular trafficking. Media collected from treated cells were centrifuged at 500*g* for 10 minutes at 4°C to remove debris. Supernatants were transferred to new tubes and centrifuged again at 3,000*g* for 10 minutes to remove smaller debris. EVs were pelleted from supernatants at 16,000*g* for 30 minutes at 4°C using an Eppendorf centrifuge 5418. The resulting EV fraction was washed with sterile PBS and recentrifuged at 16,000*g* (30 minutes, 4°C) prior to performing further analyses (120).

### Statistics.

For evaluation of SomaScan studies, data quality control, signal calibration, hybridization control normalization to remove individual sample variance, median signal and normalization to remove intersample plate differences, and calibration for interplate differences based on the pooled serum samples included on each plate were done according to the manufacturer’s protocol to correct for technical and batch effects in data introduced during the sample processing. Values that were ≥1.5 times the length of the box away from either the lower or upper quartiles of the data set for each analyte were identified and removed as outliers. All samples passed the SomaLogic standard quality control and normalization criteria. Data sets included 10 samples from patients with gastric cancer and no VTE, 10 samples from patients with gastric cancer and VTE, 10 samples from patients with non–small cell lung cancer and no VTE, and 9 samples from patients with non–small cell lung cancer and VTE. A 2-tailed *t* test was applied to evaluate the significance of differences between means of VTE versus non-VTE values for each cancer type. *P* values were corrected for false discovery rate using a Bonferroni correction factor. Analytes with a corrected *P* < 0.05 and a ≥2-fold difference between in mean values between the VTE and non-VTE groups were selected. ROC curves and 2-tailed *t* tests were performed for individual analytes. For cell culture samples, data were analyzed by 1-way ANOVA with Dunnett’s or Turkey’s post hoc multiple-test analysis, and analyses were performed using GraphPad Prism 9.1.2 software. A *P* value of less than 0.05 was considered statistically significant. Data were represented as mean ± SEM unless otherwise stated. For UPR analyses involving plasma samples, a ROC curve was performed to generate an AUC for individual UPR protein candidates. The *Z* statistic was calculated to test the null hypothesis that the AUC equals 0.5 (SigmaStat). Spearman’s rank correlation coefficient tested the association between plasma EVTF and UPR markers.

### Study approval.

The study was conducted with the approval of local ethics committees (Comitato Etico della Provincia di Bergamo, del. 146), and written informed consent was obtained from all participants.

### Data availability.

Supporting data are in the Supporting Data Values file.

## Author contributions

RF and JZ conceived the study. OM, SS, and EED developed methodology. OM, RP, CGP, and EED investigated. MY visualized data. AF, MM, and LR performed clinical study design and supervised sample collection and handling. RF and JIZ acquired funding. RF and OM wrote the original draft. RF, OM, MY, SS, and JIZ reviewed and edited the draft.

## Supplementary Material

Supplemental data

Supporting data values

## Version 1 08/31/2023

In-Press Preview

## Version 2 10/09/2023

Electronic publication
