# Supplementary material for: The unfolded protein response links ER stress to cancer-associated thrombosis
Source: JCI Insight. 2023 Aug 31;8(19):e170148. doi: 10.1172/jci.insight.170148 (PMC10629814; doi:10.1172/jci.insight.170148)
Supplement: Supplemental data [file jciinsight-8-170148-s023.pdf]

Supplemental Materials

The unfolded protein response links ER stress to cancer-associated thrombosis

Oluwatoyosi Muse, Rushad Patell, Christian G. Peters, Moua Yang, Emale El-Darzi, Sol Schulman, Anna Falanga, Marina Marchetti, Laura Russo, Jeffrey I. Zwicker, Robert Flaumenhaft

Supplemental Methods . . . . p. 2

Supplemental Table S1 . . . . .p.7

Figure S1 . . . . p. 8

Figure S2 . . . . p. 9

Figure S3 . . . . p. 10

Figure S4 . . . . p. 11

Figure S5 . . . . p. 12

Figure S6 . . . . p. 13

Supplemental Methods

Reagents

UPR inducers, tunicamycin, triptolide, MG-132, thapsigargin, the IRE1α inhibitor MKC-3946, and the PERK inhibitor GSK2606414 were obtained from Millipore Sigma. Brefeldin-A and Golgicide-A were purchased from Cell Signaling Technologies and Millipore Sigma, respectively. The human pancreatic cell line BXPC3 was purchased from Millipore Sigma. Monoclonal XBP-1s mouse IgG (D2C1F), monoclonal PERK rabbit IgG (C33E10), and GAPDH monoclonal rabbit IgG (14C10) antibodies were purchased from Cell Signaling Technologies. Monoclonal anti-human tissue factor mouse IgG1-FITC conjugated (VIC7) and monoclonal anti-human tissue factor mouse IgG (IIID8) antibodies were purchased from BioMedica Diagnostics. CD9 monoclonal mouse (5G6) antibody was purchased from Novus Biologicals. ARF1 polyclonal rabbit IgG antibody was purchased from Invitrogen. PE-Annexin V was purchased from BD Pharmingen. Human Factor X, human Factor VIIa, and Boc-L-FPR-ANSNH-C2H5 (SN-20) fluorogenic thrombin substrate were purchased from Haematologic Technologies. Alexa Fluor 488-labeled donkey anti-mouse IgG was purchased from Abcam. Alexa Fluor- 555 phalloidin and lipofectamine RNAiMAX transfection reagent were purchased from ThermoFisher Scientific. siGENOME human XBP1 siRNA, set of 4 (D-009552-01, D-009552-02, D-009552-03, D-009552-06), siGENOME Human EIF2AK3 siRNA (D-004883-02, D-004883-07), siGENOME Non-Targeting siRNA Pool #2 (D-001206-14-05), siGENOME human ARF1 siRNA, set of 4 (D-011580-01, D-011580-02, D-011580-03, D-011580-04), were purchased from Dharmacon.

Cell culture

HPAF-II were cultured in EMEM medium (Quality Biologicals) while BxPC3 cells were cultured in RPMI 1640 medium (Gibco) supplemented with penicillin/streptomycin/L-glutamine and 10% fetal bovine serum (FBS). For cell surface thrombin generation experiments, 1 X 10^4 cells were incubated in culture media for 48 h prior to experiment. For EV tissue factor activity experiments, 3 X10^4 were incubated for 48 h, followed by EV isolation. For UPR induction, cells were exposed to either tunicamycin 2.5 µg/mL,

triptolide 0.2 μM, MG132 10 μM, or thapsigargin 0.8 μM for 4 h. For studies with antagonists, cells were pretreated with UPR and ER-golgi trafficking inhibitors (IRE1α inhibitor MKC3946 5 μM, PERK inhibitor GSK2606414 1 μM, brefeldin-A 3μg/mL, or golgicide-A 1.5 μM) for 1 h prior to adding UPR inducers. siRNA transfections was accomplished using manufacturers protocols (Dharmacon). Cells were cultured at 37°C with 5% CO2.

Thrombin generation

Frozen pooled plasma derived from an average of eight healthy donors was generated by centrifuging platelet rich plasma (PRP) twice at 2800 *g* for 10 minutes before freezing at -80°C. Platelet-free plasma (PFP) was thawed at 37°C for 3 minutes. Plasma mix was prepared using 80% plasma volume, 20% HEPES buffered saline solution (HBS pH 7.4) and 0.01% GPRP (stock 500 mM). The plasma mix was added to EVs and mixed by pipetting. Substrate solution was prepared in PBS by adding 50 μM of fluorescent thrombin-specific substrate (Haematologic Technologies SN-20 Boc-L-FPR-ANSNH-C2H5) and 12.5 mM of EDTA. EV samples were then plated and mixed with 3 mM $CaCl_2$ followed by substrate solution (1:3 substrate to EV solution respectively). Thrombin activity was measured using a Synergy 4 Fluorescent Plate Reader (BioTek) for 1 hour. Excitation was set to 352 nm and emission, which was measured as a function of time, was set to 470 nm. To measure cell surface thrombin activity, plasma mix was added to cell surface after removal of media and washing with PBS to remove access media. Calcium was added at 2 mM prior to addition of substrate solution, and thrombin activity was measured. Thrombin activity was calculated based on the quantification of Vmax.

FXa generation on cell surface

5 X 10^3 cells were incubated for 48 hours after which cells were treated with tunicamycin at a final concentration of 2.5 μg/mL. Afterwards, media was aspirated and cells were washed with 200 μl buffer (HBS containing 5 mM $CaCl_2$ plus 1 mg/mL BSA). Assay buffer containing 145 nM factor X, 1.5 nM $CaCl_2$,

100 μM Factor Xa Chromogenic Substrate (biophen-CS11) was then added to cells. The FXa reaction was initiated by adding factor VIIa (Haematologic Technologies) at a final concentration of 0.1 nM diluted into assay buffer. FXa enzymatic activity was read at 405 nm once each minute for 2 hours using Spectra A max 340 PC. All experiments were performed at 37°C.

Western blotting on cell and EV lysates

Cells were seeded in 6-well plates at a density of 2×10^5 cells per well and cultured for 48 h. Following incubation with treatments for the indicated times, media was collected to isolate EVs. EVs and cells were lysed with 1X RIPA buffer (Cell Signaling) containing protease/phosphatase inhibitor cocktail mix and allowed to incubate in ice for about 5 minutes. EVs were then vortex for 2 seconds. After centrifugation, cell lysates was transferred into a new tube. For western blots, 9 μl of the lysates were mixed with 3 μl of 4x reducing sample buffer (Bio-Rad) containing β-mercaptoethanol and heated at 90 °C for 10 minutes. Samples were separated on 4–15% polyacrylamide gels (Bio-Rad), and transferred to PVDF membranes using the Trans-Blot Turbo transfer system (Bio-Rad). Membranes were blocked in 5% BSA in TBS-T. Proteins of interest were detected using primary and secondary antibodies indicated in the reagent section and allowed to incubate 1 h prior to washing. Specific protein bands on the blots were visualized by applying Clarity Max Enhanced Chemiluminescence western blotting substrates (Bio-Rad) and then recorded with Bio-Rad ChemiDoc MP imaging system. Band intensity was quantified using Bio–Rad Image lab touch software.

Immunogold-electron microscopy of cells and EVs

Cells were removed from 6-well plates with accutase. 800 μl of the cell suspension was then layered on top of a 200 μl cushion of 4% paraformaldehyde plus 0.1% glutaraldehyde (in 0.1M sodium phosphate buffer, pH 7.4) in an Eppendorf tube and pelleted for 3 minutes at 3000 rpm. Supernatant was carefully removed and fresh 4% paraformaldehyde plus 0.1% glutaraldehyde added. After 2 h fixation at room temperature the pellet was washed once with PBS and then placed in PBS containing 0.2 M glycine to

quench free aldehyde groups for 15 min. After incubation cells were washed once with PBS. For EV preparation, EVs were first isolated as described above and the same fixation steps were followed. Subsequent steps such as freezing, gold labeling, primary and secondary antibody staining and imaging were performed at the Harvard Medical School electron microscopy facility.

Immunofluorescence of HPAF-II cells

Cells were seeded on cover slips in a 12 well plate until they were 40% confluent. After treatments with UPR inducers, cells were washed with PBS to remove remaining media. Cells were fixed with 4% Paraformaldehyde (PFA) solution for 15 minutes. Cells were then washed with PBS 3X followed by blocking buffer (45 ml of PBS, 5 ml of Goat serum, .5 grams of BSA) for 30 minutes. For permeabilization, cells were incubated with 0.25% Triton X-100 in PBS for 15 minutes, then washed 3X with PBS. Primary and secondary antibodies were prepared in blocking buffer and allowed to incubate for 1 h with 3X PBS washes between primary and secondary antibody treatments. For nuclear staining, cells were treated with DAPI (0.5 µg/ml) in PBS for 10 minutes then washed 2X with PBS. Using a basic mounting protocol, cover slips were prepared for imaging. Imaging was performed on a Zeiss LSM 880 Upright Laser Scanning Confocal Microscope. To quantify tissue factor expression levels, the green channel was parsed out in ImageJ and the fluorescence intensity was extrapolated after background subtraction from an area without any cells. Fold-increases were calculated from the tissue factor fluorescence compared to the control. Frequency of cell blebs was determined by counting peripheral cells since these were the only cells that could be analyzed for blebs. Nuclei of these cells were quantified for total cell counts. A cell was determined to be blebbing if following uniform thresholding there was TF staining visible beyond the F-actin staining. Cells with no tissue factor or colocalized signal were determined to be negative for blebbing. Data represent blebs/nuclei as determined for the indicated number of slides and presented as a percentage.

Flow cytometry

Levels of tissue factor protein and phosphatidylserine (PS) on the surface of cell derived EVs were measured using flow cytometry. Cell-derived EVs were suspended in 100 µL PBS, to which monoclonal anti-human tissue factor mouse IgG, FITC conjugated 10 µg was added and allowed to incubate for 30-60 minutes. As a negative control, Mouse IgG1 isotype was used. After incubation, EV solution was spun down, solution removed and PBS added for wash (wash as needed). Subsequently, annexin V staining buffer (Abcam) and PE annexin V (per manufacturers protocol) were added to EV pellets and allowed to incubate for 10 minutes prior to imaging. Flow experiments were performed using Beckman Coulter MoFlo Astrios EQ cytometer and FlowJo analysis software was used to process the data. EVTF measurement in plasma was previously performed utilizing an impedance-based flow cytometer (Beckman Coulter SC Quanta) and labeled using humanized monoclonal antibodies cH36 against human TF *(25, 35)*.

Real-time PCR

Real-time PCR was performed using the TaqMan™ Gene Expression Cells-to-CT™ Kit according to the manufacturer's instructions, with Human GAPDH (VIC-MGB) and Human Tissue Factor (FAM-MGB) used as primers to evaluate gene expression. Applied Bio-system 7300 Real-Time PCR system and software were used for data collection and analysis.

Table S1. Patient Characteristics.

	Total (n=39)	VTE (n=19)	No VTE (n=20)
**Patient Demographics and Co-morbidities**			
Age, median (IQR)	65 (58,74.5)	62(57.5,70.5)	66.5 (61.75,75.5)
Female sex	20	9	11
Weight, median (IQR)	60 (49-72.5)	66 (50-72.5)	60 (47.75-73)
BMI, median (IQR)	20.72 (16.85-24.95)	22.65 (20-26.95)	20.41 (11.7-22.9)
Prior history of VTE	2	2	0
Antiplatelet agent use	4	1	3
**Cancer Site**			
Non-small cell lung	19	9	10
Gastric	20	10	10
**Metastatic Disease**			
Yes	39	19	20
No	-	-	-
**ECOG Score**			
0	14	9	5
1	31	9	12
≥2	4	1	3
**Khorana Score**			
<2	17	10	7
≥2	22	9	13
**Thrombotic Events**			
Time to Thrombotic Event, median (IQR) (months)	2 (2-4)	2 (2-4)	-
Pulmonary Embolism	1	1	-
**Deep Vein Thrombosis**	13	13	-
Symptomatic	11	11	-
Asymptomatic	2	2	-
Lower Extremity	8	8	-
Upper Extremity	4	4	-

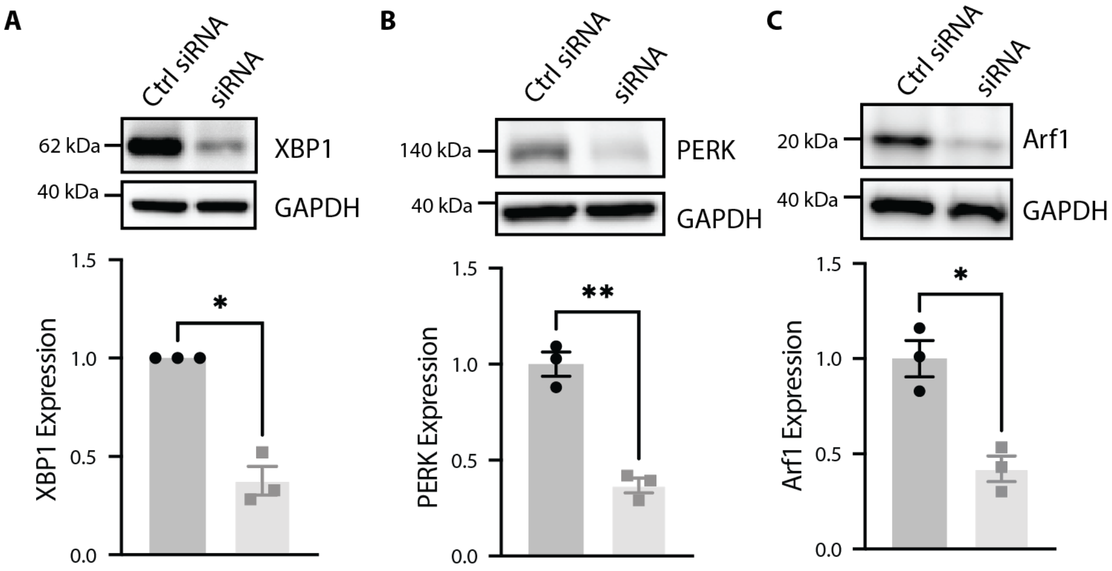

Figure S1. Western Blot evaluation of siRNA knockdowns. HPAF-II cells were transfected with non-targeting siRNA (control) or siRNA targeting human (**A**) *XBP1* (**B**) *PERK* or (**C**) *ARF1* at a concentration of 40 nM for 48 hours then re-inoculated with 40 nM of siRNA and allowed to incubate for 24 hours. After siRNA treatment, media was replaced and cells were treated with 2.5 μg/mL tunicamycin for 4 hrs. Cell lysates were analyzed by immunoblotting using anti-PERK, anti-XBP1, and anti-ARF1 antibodies. GAPDH was used as a loading control. Graph represent band intensities as quantified from Image J software. Each data set represents the mean$\pm$SEM of 3 samples. (**P<0.001, *P<0.02, *P<0.01 for PERK, XBP1 and ARF1).

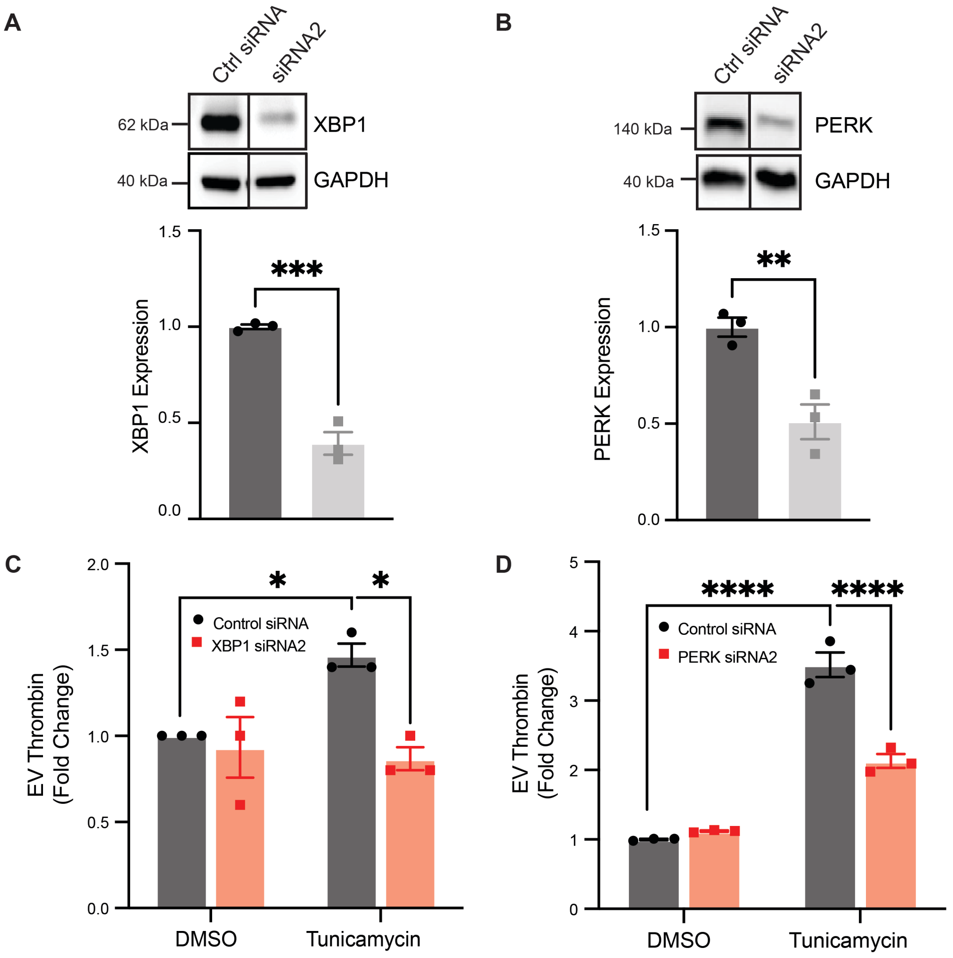

Figure S2. Confirmation that knockdown of IREα/XBP1 or PERK pathway results in decreased UPR-induced generation of procoagulant EVs. (**A**) HPAF-II cells were exposed to 40 nM of either control siRNA or siRNA (a second siRNA directed at XBP1, distinct from the siRNA used in Figure 3) for 72 hours and subsequently exposed to either DMSO or 2.5 μg/mL tunicamycin for 72 hours. EVs were isolated from supernatants and evaluated for thrombin generation. Error bars represent the mean±SEM of 3 samples, **p<0.01. (**B**) HPAF-II cells were exposed to 40 nM of either control siRNA (a second siRNA directed at PERK, distinct from the siRNA used in Figure 3F) for 72 hours and subsequently evaluated as described in (**A**). EVs were isolated from supernatants and evaluated for thrombin generation. Error bars represent the mean±SEM of 9 samples, *p=0.01.

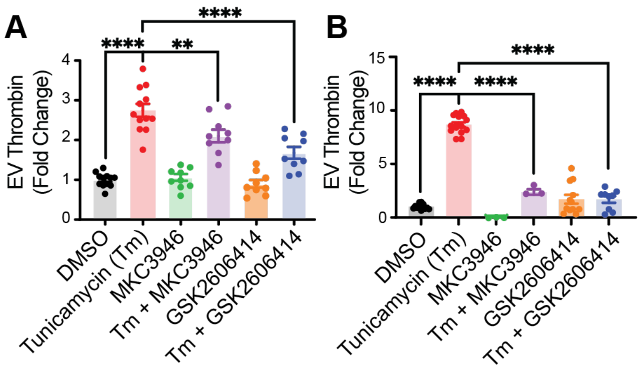

Figure S3. Inhibition of UPR impairs the generation of procoagulant EVs from gastric and lung adenocarcinoma cells. (**A**) AGS gastric carcinoma and (**B**) A549 lung carcinoma were incubated with either 5 μM IRE1α inhibitor MKC3946 or 1 μM of PERK inhibitor GSK2606414 for 45 minutes followed by 2.5 μg/ml tunicamycin for 4 hours. Supernatants were collected and EVs analyzed for thrombin generation.

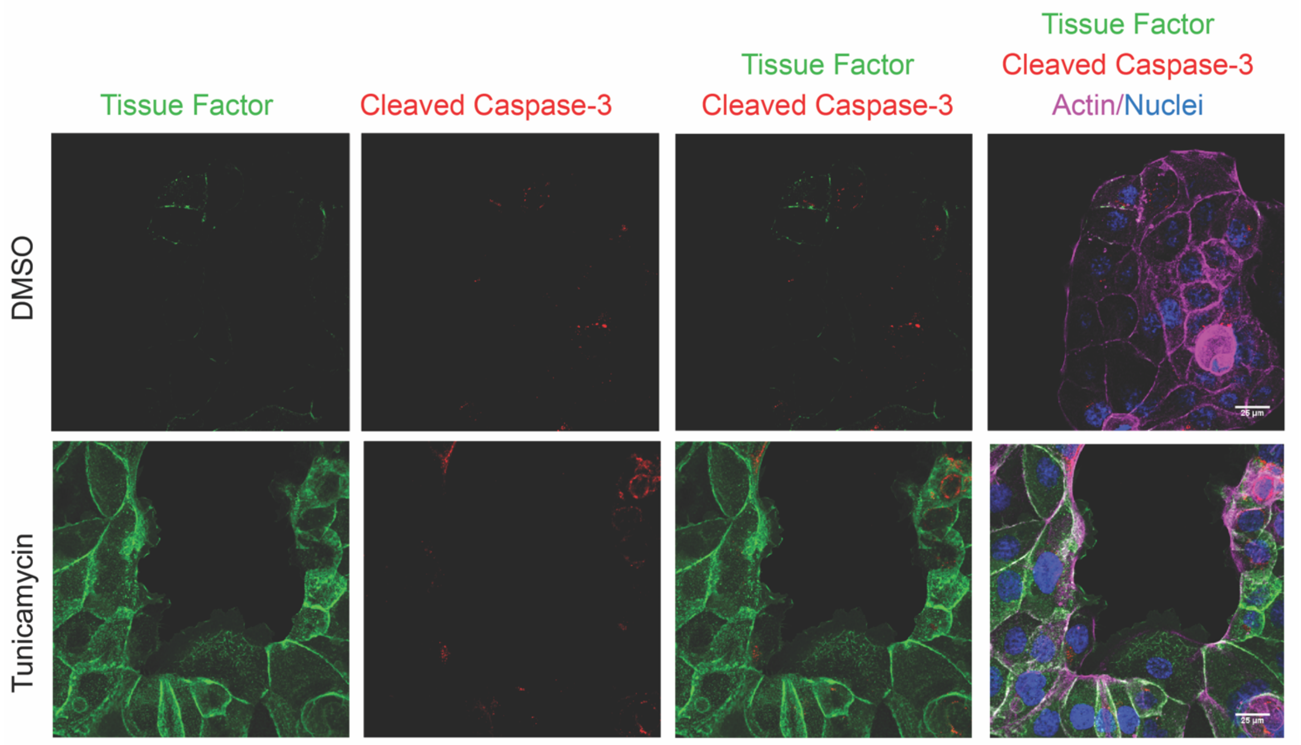

Figure S4. Immunofluorescence detection of apoptosis in HPAF-II treated cells. HPAF-II cells were treated with 2.5 µg/mL of tunicamycin or DMSO for 4 hrs, washed in PBS, and fixed with 4% PFA. Tissue factor (*green*), cleaved caspase 3 (*red*), actin (*purple*) and cell nuclei (*blue*, DAPI) were imaged using confocal immunofluorescence microscopy.

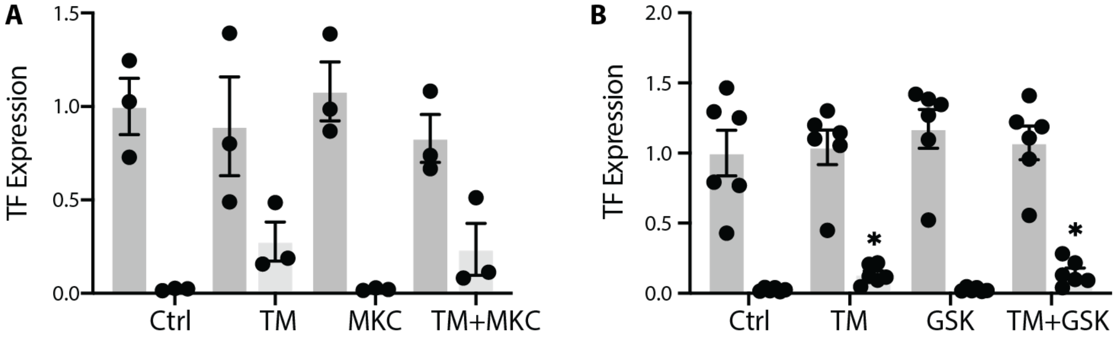

Figure S5. Quantitative analyses of IRE1α and PERK inhibitors on TF expression in HPAF-II cell lysates. HPAF-II cells were pre-incubated with (**A**) MKC3496 (5 μM) and (**B**) GSK2606414 (1 μM) prior to stimulating with tunicamycin and tissue factor detected in lysates as described in Figs. 6B and 6C. Data represents the mean$\pm$SEM of 3-6 samples; dark grey bar indicates upper band and light grey bar indicates lower band.

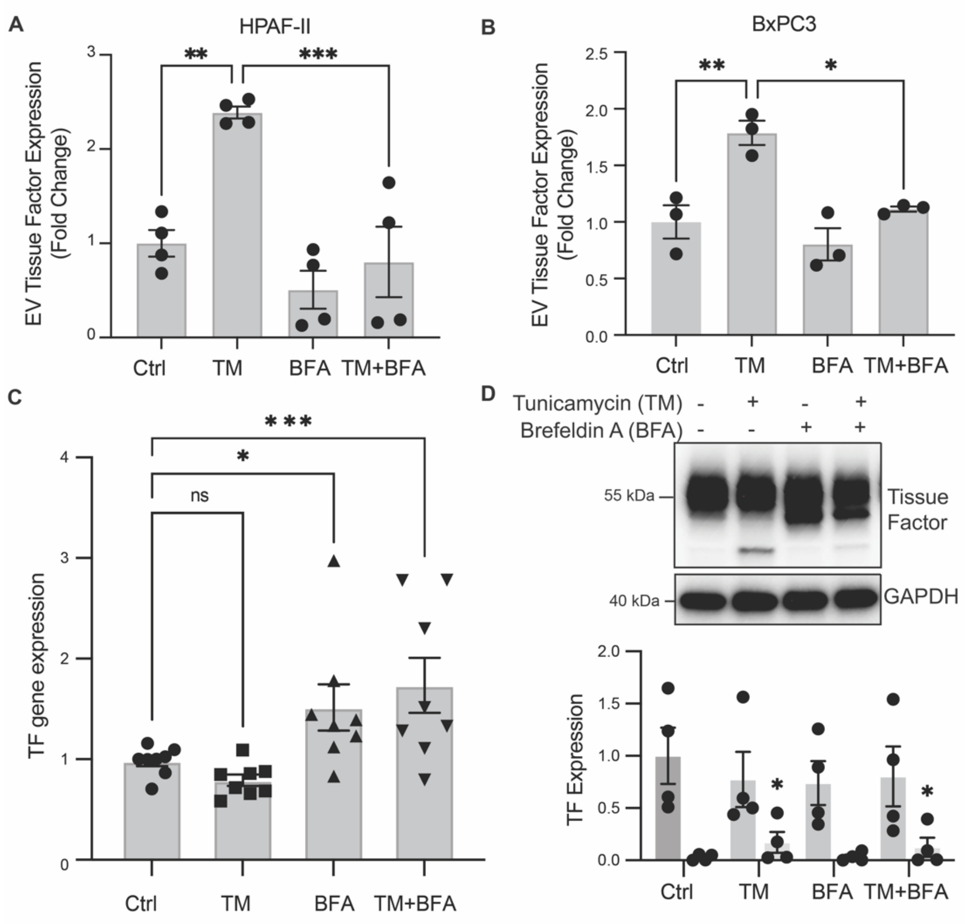

Figure S6. Brefeldin A inhibits trafficking of TF to extracellular vesicles. (**A**) HPAF-II cells or (**B**) BxPC3 cells were incubated in the presence of vehicle (DMSO) or brefeldin A prior to stimulation with tunicamycin as described in Fig. 6A and 6B. EVs were collected and evaluated for TF by Western blot analysis. Levels of TF positive extracellular vesicles were normalized to control and plotted as fold change. Data represents the mean±SEM of 3-4 samples. (*P<0.02, **P<0.01, ***P<0.0005) **(C)** HPAF-II cells were incubated with brefeldin-A (3 μg/mL) for 45 min prior to addition of tunicamycin (2.5 μg/mL) as indicated. TF mRNA was then quantified using RT-PCR. Data represent the mean±SEM of 8 samples. (*P<0.03, ***P<0.001, one-way Anova with Turkey post-hoc). **(D)** HPAF-II cells were preincubated with brefeldin-A (3 μg/ml) for 45 minutes prior to stimulating with tunicamycin (2.5 μg/mL) for 4hrs. TF in whole cell lysates was analyzed by western blot analysis. GAPDH was used as a loading control. Glycosylated (*upper band*) and deglycosylated (*lower band*) TF were analyzed separated. Lower graph represents band intensities. Data set represents the mean ±SEM of 4 samples (*P<0.02, paired t-test).
